# Bilateral Fitting of Bone-Anchored Hearing Devices: Speech Recognition and Sound Localization

**DOI:** 10.1055/s-0045-1810002

**Published:** 2025-10-16

**Authors:** Cynthia Harumi Yokoyama Ueda, Isabela de Souza Jardim, Ricardo Ferreira Bento, Renata Marcial Soares

**Affiliations:** 1 Department of Otorhinolaryngology, Hospital das Clínicas, School of Medicine, Universidade de São Paulo, São Paulo, SP, Brazil

**Keywords:** bilateral hearing loss, hearing aids, bone-anchored prosthesis, bone conduction, correction of hearing impairment, speech discrimination tests

## Abstract

**Introduction:**

Bilateral fitting of bone-anchored hearing devices (BAHD) remains a contentious topic, as the use of a single device can direct sound to both cochleae. However, the interplay of interaural attenuation and time delay may result in different responses in each ear; thus, greater challenges for unilateral BAHD users.

**Objective:**

To assess the auditory performance of BAHD users with unilateral and bilateral fitting regarding speech recognition in silence and noise and sound localization.

**Methods:**

Cross-sectional study. Unilateral BAHD users treated at a Brazilian public hospital underwent a speech recognition test, containing a list of monosyllables at 65dBSPL in silence and noise (S/N ratio = 0dB). The sound localization test employed eight speakers arranged at 45° distance. A recorded trisyllabic word served as the stimulus. For bilateral fitting, an elastic band with a device similar to the implant was used; patients underwent the same tests under identical conditions.

**Results:**

Sixteen patients were assessed (mean age = 24.2 years). The average monosyllable recognition in silence rate was 71.5% in unilateral fitting and 78.2% in bilateral fitting. In noise, 61% in unilateral fitting and 72.2% in bilateral fitting. The mean angular deviation for the sound localization test was 80.48° in unilateral fitting and 68.08° in bilateral fitting. The bilateral fitting outcomes were better for all tests.

**Conclusion:**

Users with bilateral BAHD fitting had better outcomes for speech recognition and sound localization.

## Introduction

The bone-anchored hearing device (BAHD) converts acoustic sound waves into mechanical vibration, which is transmitted to the inner ear through direct contact with the skull. BAHD can be classified into percutaneous or transcutaneous devices.

The currently available percutaneous devices include the Ponto™ (Oticon Medical) and the Baha® Connect (Cochlear Co). The Baha® Attract System (Cochlear Co) and the Alpha 2 MPO™ (Medtronic) are passive transcutaneous devices. The active transcutaneous devices include the Bonebridge (MED-EL) and the Osia® 2 (Cochlear Co).

The Ponto™ and Baha® devices can be tested before surgery using a softband adjusted on the patient's head. Due to the presence of skin and subcutaneous tissue, sound transmission is dampened, resulting in lower effective amplification. However, the softband is considered to be the best pre-surgery testing method once it is the closest to the implant result, with a difference of 1 to 13dB 1 and the minimal clinical distinction between pre-surgery and post-activation testing. 2 3 4 The softband is also used to assess bilateral BAHD in sequential use, which is when the patient already uses the device on one side and undergoes further testing to evaluate the benefits of bilateral adaptation. 5

Since both cochleae receive stimulation in a unilateral BAHD fitting, the bilateral fitting in patients with bilateral conductive or mixed hearing loss is controversial. However, interaural attenuation of bone conduction is not always null and can vary between 0 to 15dB. 6 Hence, it cannot be asserted that both cochleae receive identical stimulation from a unilateral bone-conduction stimulus.

In addition to interaural attenuation, there is also a interaural time difference for the sound to reach the contralateral cochlea, which varies between 0.3 to 0.5ms for frequencies above 1000Hz. Considering the co-occurrence of interaural attenuation and interaural time difference, the ipsilateral and contralateral bone-conducted signals will not result in identical cochlear responses, potentially leading to different responses in both ears. 7 This may contribute to greater challenges in terms of sound localization and speech discrimination in noisy environments, as well as an increased listening effort for individuals using a unilateral BAHD.

Some studies show various advantages in the bilateral BAHD fitting compared to the unilateral, 8 9 10 11 12 13 such as improved speech perception in silence and most noise conditions, better sound localization and lateralization, better sound detection, and improved quality of life assessed with self-assessment questionnaires.

There are few studies in the literature with good quality evidence using objective assessment methods, and usually, they present a small sample size. The stimulus used to assess the speech recognition performance of BAHD users varies. Most identified studies use sentence lists in their language. To the best of our knowledge, there are no Brazilian studies regarding the bilateral fitting of BAHD.

As technologies continue to advance, new auditory devices are continually entering the market to enhance sound quality and minimize the inherent risks associated with implant surgery. This highlights the importance of up-to-date studies on the real cost-benefit of bilateral implantation and fitting grounded in objective evaluations. Therefore, the present study aimed to assess the auditory performance of BAHD users with unilateral and bilateral fitting regarding speech recognition in silence and noise, and sound localization.

## Methods

### Study Population

This cross-sectional study was carried out at a tertiary public hospital in Brazil. The research was approved by the Research Ethics Committee under protocol number 4,254,874.

Participants included both male and female patients who had undergone implantation. All patients had met the criteria of the Brazilian Ministry of Health 14 which only approves a unilateral BAHD.

Exclusion criteria included:

- Patients implanted with technologies that were incompatible with softband testing;
- BAHD short period of usage (less than 8 hours a day);
- Phonological disorder.

At the time the present study was performed, the processors utilized for percutaneous devices within the Brazilian Unified Health System (SUS) included the Ponto 3 generation (Oticon Medical AB) and the Baha 5 (Cochlear Co).

### Procedures

The procedures were conducted in a single session following the sequence:

a) Evaluation with unilateral fitting, using the device itself, connected to the abutment, including speech recognition in silence and noise and the sound localization test;
b) Fitting of a similar contralateral device with the assistance of the softband;
c) Device fitting via brand-specific software after performing *in situ* bone conduction audiometry;
d) Evaluation with bilateral fitting (implanted device + contralateral softband) was performed using the same speech recognition test in silence and noise, and the sound localization test.

### Speech Recognition Tests

The Itera II Audiometer was employed within an acoustic booth fitted with a free-field system. The patient was positioned 1.0 meters away with the head angle set at 0° azimuth from the speaker.

A list of 25 phonetically balanced monosyllabic recorded Brazilian Portuguese words 15 was presented at 65dBSPL. Speech Noise (SN) served as competitive noise that was presented in the same intensity; the signal-to-noise ratio (S/N) was 0dB. Both signal and noise were presented through the same speaker at a 0° azimuth (S _0_ N _0_ ). The patients were instructed to repeat the words out loud.

The results are expressed as percentages. The speech recognition outcomes considered the answers obtained with unilateral and bilateral BAHD adaptation.

### Sound Localization

The sound localization assessment was conducted in the *Laboratório de Pesquisas de Habilidades Auditivas* (LHAB), freely translated as Auditory Skills Research Laboratory. The LHAB room dimensions are 3.70m (width) x 3.98m (length) x 2.10m (height). The LHAB has eight speakers equally distributed at 45° angular intervals. The audio control software employed was Reaper (Cockos Incorporated, 2004, USA).

The patient was positioned at the center of the eight speakers. The speakers were visibly numbered from 1 to 8. The distance from the speakers to the center, where the patient was positioned (radius), was 1.20 meters.

The stimulus used was a trisyllabic Brazilian Portuguese word, recorded in a male voice. The chosen word was “cadeira" (chair). This portuguese word was chosen due to its proximity to the frequency range of 500 to 3000Hz.

Initially, a training session was performed. The word was presented at each speaker sequentially, from 1 to 8. Following the training, the stimulus was randomly presented three times to each speaker. The patient was asked to identify the stimulus sound source by pointing and/or stating the corresponding speaker number.

The result is a mean angular deviation calculated based on the patient's 24 responses. Responses were computed or registered as the angle difference from the correct localization (angular deviation). For example, if the stimulus was presented at speaker 1 and the patient responded to speaker 2 or speaker 8, the deviation was 45°; if the response was speaker 3 or speaker 7, the deviation was 90°; if the response was speaker 4 or speaker 6, the deviation was 135°; if the response was speaker 8, the deviation was 180°. If the patient answered correctly, the deviation was 0°.

**Fig. 1:**
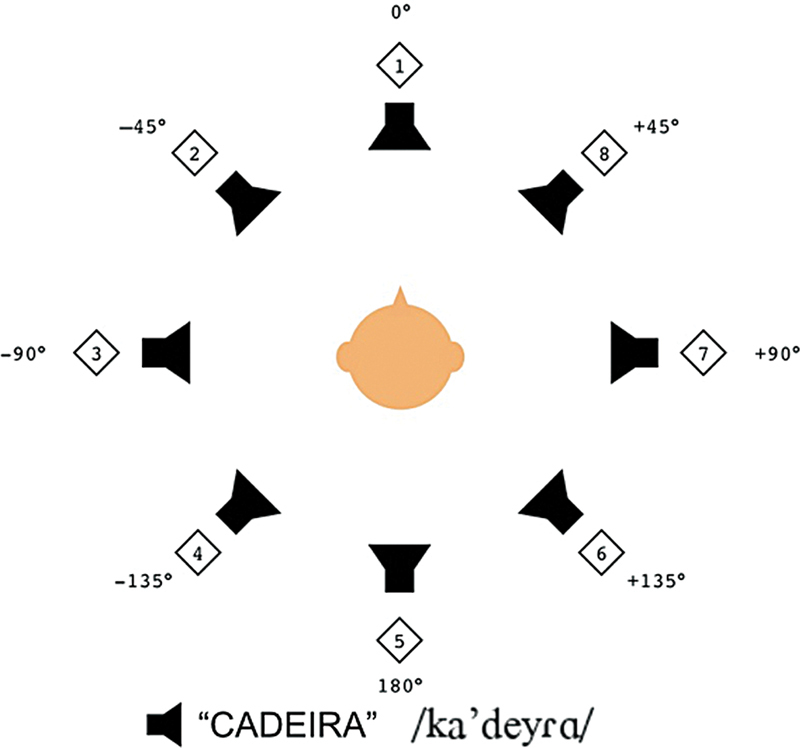
Speakers distribution in the LHAB and the phonological transcription of the stimuli.

Figure 1 shows the speakers distribution in the LHAB and the phonological transcription of the stimuli “cadeira”.

### Statistical Analysis

The objective of the statistical analysis was to investigate the effect of uni- or bilateral fitting and the silent and noisy conditions, as well as the interaction between these factors, on the percentage of monosyllable identification. Individuals were inserted as random effects of the intercept of the model, since there was significant variance between the intercepts according to the individuals (intraclass correlation coefficient (ICC) = 0.542, χ ^2^ (1) = 21.379, p < 0.001, Likelihood Ratio Test (LRT)). Fitting and condition were entered as fixed effects. The significance of the fixed effects was assessed with F tests with degrees of freedom calculated using the Kenward-Roger method and effect size calculated by converting the F statistic to the r coefficient (Field, 2017). The variance components were estimated using the Residual Maximum Likelihood (REML) technique and an “unstructured” covariance structure was assumed.

For investigate the effect of uni or bilateral fitting over the angular deviation mean on sound localization test, a linear mixed model (Linear Mixed Model – LMM) was developed. Individuals were inserted as random effects of the model intercept, since there was significant variance between the intercepts depending on the individuals (intraclass correlation coefficient (ICC) = 0.542, χ ^2^ (1) = 21.379, p < 0.001, ratio test likelihood [Likelihood Ratio Test – LRT]). Unilateral or bilateral fitting was entered as fixed effects.

## Results

A total of 38 implanted patients were initially selected. Among these, 15 were excluded once they were Bonebridge users and this device is incompatible with testing using the softband. Of the remaining 23 patients, two were excluded due to ineffective use of the device; three presented phonological disorder; one patient could not be contacted and one patient presented visual impairment, preventing the sound localization test.

Table 1 presents the sample characterization.

**Table 1:** Sample Characterization

Patient	Sex	Age (years)	Implanted Side	Device	Surgery (months)	Activation (months)
1	M	73.0	L	PONTO 3 SP	3.5	2.6
2	F	47.1	R	BAHA 5 POWER	12.8	11.5
3	F	24.6	R	PONTO 3 POWER	46.5	44.9
4	F	56.5	R	BAHA 5 POWER	9.5	7.5
5	M	21.4	R	BAHA 5	121.2	118.0
6	M	20.6	L	PONTO 3 SP	34.3	30.9
7	F	16.0	L	BAHA 5	26.4	16.3
8	F	9.9	R	BAHA 5	21.9	17.2
9	F	14.6	R	PONTO 3 SP	44.4	41.5
10	F	19.7	L	BAHA 5 POWER	57.6	49.5
11	F	13.7	L	PONTO 3 SP	53.2	48.1
12	F	18.6	R	PONTO 3 SP	59.8	6.44
13	M	12.5	L	BAHA 5 POWER	17.0	12.6
14	M	10.1	L	BAHA 5	2.0	0.2
15	M	16.2	L	BAHA 5	34.3	30.9
16	F	12.6	R	BAHA 5	44.1	41.4

Legend: F, female; L, left; M, male; R, right.

Table 2 displays the measures of central tendency and dispersion for the sample characterization concerning age, activation time, and surgery time.

**Table 2:** Sample characterization regarding age, activation, and surgery

Variable	n	Mean	SD	Min.	Max.
Age	16	24.20	18.31	9.82	72.99
Activation (months)	16	29.99	29.09	0.23	118.05
Surgery (months)	16	36.79	29.35	2.04	121.17

Legend: Max., Maximum; Min., Minimum; SD, Standard Deviation.

### Speech Recognition

In silence, the average monosyllable recognition score in unilateral fitting was 71.5% (SD = 11.8, with a minimum of 52% and a maximum of 96%); in bilateral fitting, the recognition score was 78.2% (SD = 13.2, with a minimum of 56% and a maximum of 96%).

In noise, the average recognition score in unilateral fitting was 61% (SD = 11.7, minimum of 36% and maximum of 84%); in bilateral fitting, this score was 72.2% (SD = 15.3, minimum of 48% and maximum of 92%).

**Fig. 2:**
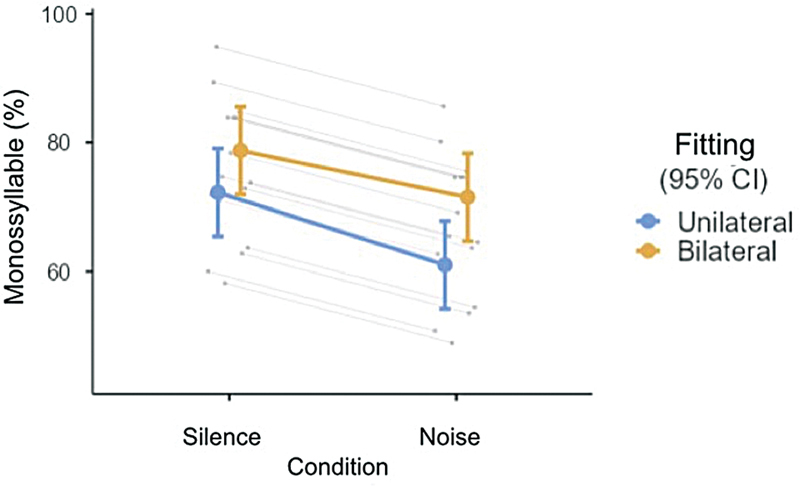
Estimated marginal means of monosyllable recognition percentage according to fitting and condition. Error Bars = 95% Confidence Interval Note: Primary and secondary lines represent data for the total sample and for each patient, respectively.

The fixed-effects test presented significant effect of noise presence (F (1,45) = 29.804, p < 0.001, r = 0.631) and fitting (F (1,45) = 25.167, p < 0.001, r = 0.599); however, there was no significant interaction between these factors (F (1,45) = 1.393, p = 0.244, r = 0.173), on the percentage of monosyllable recognition in silence and in noise. Hence, despite the presence of noise, the bilateral fitting presented a higher recognition score for monosyllables compared to unilateral fitting (mean difference = 8.50, CI 95% *bootstrap* = [5.18, 11.70], t = 5.017, p < 0.001, r = 0.599). Similarly, regardless of the fitting, a lower percentage of monosyllable rate recognition was observed in the presence of noise (mean difference = −9.25, CI 95% *bootstrap* = [−12.67, −5.89], t = −5.459, p < 0.001, r = 0.631). ( Fig. 2 )

### Sound Localization

Table 3 presents the measures of central tendency and dispersion for the mean angular deviation in the sound localization task according to the fitting.

**Table 3:** Descriptive values of mean angular deviation in the sound localization task according to the adaptation

SOUND LOCALIZATION – MEAN DEVIATION (°)		
Adaptation	Unilateral	Bilateral
Mean	80.48	68.08
SD	17.43	19.90
Minimum	45.00	31.87
Maximum	106.87	101.25

Legend: Max.: Maximum; Min.: Minimum; SD: Standard Deviation.

**Fig. 3:**
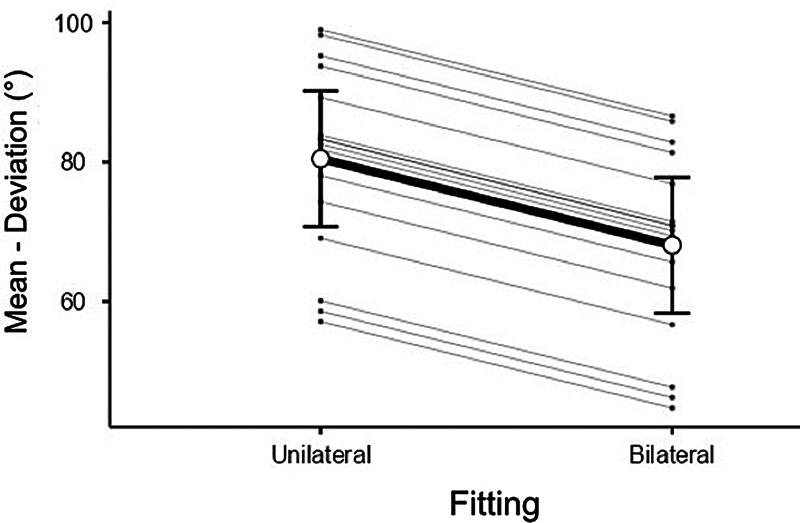
- Estimated marginal means of mean deviation in the sound localization task according to fitting. Error Bars = 95% Confidence Interval Note: Primary and secondary lines represent data for the total sample and for each patient, respectively.

The fixed-effects test presented a significant effect of the fitting (F (1,15) = 10.430, p = 0.006, r = 0.640) on the mean deviation. Therefore, a lower mean deviation was observed in the sound localization task for bilateral fitting compared to unilateral fitting (mean difference = −12.40, CI 95% *bootstrap* = [−20.16, −4.92], t = −3.230, p = 0.006, r = 0.640). ( Fig. 3 )

## Discussion

The patients selected to participate in this study were patients treated at the Bone-Anchored Hearing Devices Clinic ( *Ambulatório de Próteses Osteoancoradas* ) of a public Brazilian hospital. All implanted patients in the clinic had bilateral hearing loss. However, by SUS authorization, they underwent only unilateral surgery. Only one patient with Fraser Syndrome and significant visual impairment was authorized to receive bilateral adaptation.

Patients presenting phonological disorder were excluded, since it would not be possible to determine whether possible errors in the monosyllable test were due to these substitutions or not. Also, patients who did not report effective use of the devices were excluded. This study did not specifically investigate the reasons for non-device usage; nevertheless, it is known that, although the surgical implantation of percutaneous devices is generally easily executable and safe, 16 17 it necessitates daily cleaning and care around the abutment region. Neglecting this care can lead to complications related to pain, skin irritation, infections, and osseointegration failure. 18

It is important to highlight that all patients attending this service undergo the same protocol. Ueda et al. 19 described the medical and audiological evaluation process that all patients undergo during the implantation selection process and the follow-up post-surgery. Among the process steps, there is guidance on care and hygiene with the abutment region, in addition to instructions on the functioning, handling, and maintenance of the external component. The protocol also refers to periodic follow-ups post-surgery with predetermined returns at one, three, six- and 12-months post-surgery. After the first 12 months, the patients are advised to return each year for follow-up; however, not all patients do.

The present study counted with 16 patients, 10 women (62.5%) and 6 men (37.5%). The age varied from 9 to 73 years old, mean age = 24.20 years. While the age variation was notably high, this was not deemed negative once previous studies showcased positive outcomes and audiological benefits even within the pediatric population. Children using BAHD in the study conducted by Dun et al. 20 demonstrated effective use and a high level of satisfaction with their devices. Additionally, Roman et al. 21 reported a functional success rate of over 96% in children using BAHD, and Reis et al. 22 concluded that BAHD is an effective and safe method in auditory rehabilitation for children.

The duration of device usage also exhibited considerable variability among the participants. The patient with the shortest usage time had undergone activation less than 1 month before the assessment, while the most experienced patient had been a user for many years. This variation was not considered a limitation for the study, once previous research indicates that outcomes with BAHD are quickly evident, and patients achieve good performance even with limited usage time. A previous study conducted in the same clinic 19 characterized patients and performed audiological evaluations, including monosyllabic word recognition tests in silence; it was observed that even patients with a short usage time already demonstrated good performance in the test, highlighting the rapid onset of positive outcomes with BAHD. Catalani et al. 4 also found no difference in the performance of speech perception in noise at the time of activation and six months after. Similarly, Caspers et al. 12 found no difference in the performance of the sound localization test over time.

The objective of the present study was to determine if the auditory performance with bilateral BAHD fitting is superior to unilateral fitting regarding speech recognition in silence and noise and sound localization.

BAHD remains a contentious subject, with limited coverage in the existing literature, particularly within the Brazilian context. There is a scarcity of recent studies addressing BAHD bilateral fitying, and as far as our knowledge extends, no such study has been conducted in Brazil. Furthermore, the field is evolving with the introduction of new devices designed to enhance sound quality and mitigate the inherent risks associated with implant surgery. Thus, studies must be up to date on the real cost-benefit of bilateral implantation.

The fitting of a single bone-anchored hearing device stimulates both cochleae. In 2017, Celikgun & Kalciolgu 23 concluded that unilateral fitting may prevent or reduce neural deprivation in the contralateral ear after assessing speech recognition in silence in unilateral bone-anchored hearing device users. However, the authors agreed that employing monosyllabic words would be more suitable than trisyllabic words for making this comparison. The present study used monosyllabic words based on a previous study 19 that demonstrated greater consistency in the patient's response using monosyllables; probably because monosyllabic words provide fewer clues than longer words. Additionally, in sentence evaluations, the patient must correctly identify all words in the sentence for it to be considered a correct response.

Studies that have concluded that bilateral fitting is superior to unilateral fitting in speech recognition in silent conditions utilized sentences with a minimum of three words as stimuli. 8 9 10 24 25 Kompis et al. 26 used a list of monosyllabic words in German and observed no advantage of bilateral fitting over unilateral fitting. In contrast to the outcomes reported in the literature, the current study observed enhanced performance in bilateral fitting during the speech recognition test using monosyllabic words, a task considered to be more challenging.

Also, the present study observed that the presence of noise directly interferes with speech recognition performance. The literature shows mixed results regarding performance in speech recognition in noise. Bosman et al. 9 and Priwin et al. 10 found no significant difference between bilateral and unilateral fitting in speech recognition in the presence of noise. Snik et al., 8 Dutt et al., 24 and Priwin et al. 25 observed an advantage in bilateral fitting when noise was presented in the contralateral ear but not when the noise was presented in the same side of the implanted ear; also, there was no significant difference when signal and noise were presented in front of the patient. In contrast to these previous studies, the present study presented the advantage of bilateral fitting over unilateral in speech recognition in noise with S _0_ N _0_ condition, i.e., signal and noise coming from the same direction at 0° azimuth.

Regarding the sound localization test, all the reviewed studies observed the advantage of the bilateral fitting, per the present study. Nevertheless, in contrast to the literature, a verbal stimulus was employed instead of a noise stimulus. All the cited studies 8 9 10 12 13 25 27 used a noise stimulus, but this study used a recorded word: "cadeira" (chair). The noise bursts of the previous research varied between 500 to 3000Hz, once it is the frequency range where BAHDs have better effective amplification. The word choice "cadeira" (chair) was also because it maintains this frequency range and the belief that a verbal stimulus may better represent daily situations.

The present study's scoring methods were also another difference in the sound localization assessment. Some studies employed the Correct vs. Incorrect punctuation method. 10 13 25 Other studies considered a "good" score if the patient pointed out the correct speaker or the immediately adjacent one, at 45° or 30°, 9 depending on the number of speakers used in the research. Some studies considered accurate lateralization. 9 12 27 On the other hand, the present study, assessed the sound localization if bilateral fitting provides more auditory cues, therefore, the patient has a greater chance of locating the sound source or making a smaller error if unable to locate the exact source. Thus, we used the angular deviation analysis. Differently from what was expected, not all 16 patients presented a smaller mean angular deviation using the bilateral fitting when compared to the unilateral fitting. Two patients obtained the same mean deviation with unilateral and bilateral fitting. Other two patients presented a higher mean angular deviation with the bilateral fitting. This individual variability was controlled using a mixed linear model in the statistical analysis. Thus, a smaller mean angular deviation was observed with bilateral fitting compared to unilateral fitting, as expected.

Moreover, diverging from earlier studies, this current research concurrently assessed both fitting options - unilateral and bilateral - for the same participants. This may reduce bias regarding acclimatization or ability improvement, unlike when the patient is evaluated after undergoing surgery for the second device. This process might involve a time gap of several months, considering the recovery and osseointegration time between surgery and activation. It also helps control biases observed when compared with a control group.

In this study, bilateral fitting was attained using an softband, which, as per the literature, may yield inferior results when compared to the actual implant. 1 26 Therefore, it can be stated that the performance with the softband simulating bilateral fitting was superior to unilateral fitting and it would be expected that the results for each patient with the actual BAHD would have been even better than those obtained in this study.

It is noteworthy that the present study evaluated the BAHD audiological benefits. However, other aspects must be considered before indicating a bilateral fitting. For instance, it is important to consider complications that are inherent to the use of BAHD. While the patient may benefit from contralateral fitting in case of skin and osseointegration complications or if one device fails, the patient will undergo two surgical procedures. Also, the patients must take necessary care with the abutment or magnet regions on both sides, potentially increasing the chances of complications associated with use.

Therefore, despite the present study demonstrating the advantage of BAHD bilateral fitting, further research on these new devices available on the market is extremely important. Additionally, other aspects should be taken into consideration, consistently giving precedence to the user's final choice.

## Conclusion

The bilateral BAHD fitting outperforms the unilateral fitting regarding the abilities of speech recognition in silence, speech recognition in noise, and sound localization.
